# Supplementary material for: Counterregulation of cAMP-directed kinase activities controls ciliogenesis
Source: Nat Commun. 2018 Mar 26;9:1224. doi: 10.1038/s41467-018-03643-9 (PMC5964327; doi:10.1038/s41467-018-03643-9)
Supplement: Supplementary file 1 — Supplementary Information(PDF 14594 kb)(PDF 14631 kb) [file 41467_2018_3643_MOESM1_ESM.pdf]

Counterregulation of cAMP-directed kinase activities controls ciliogenesis

Porpora et al.

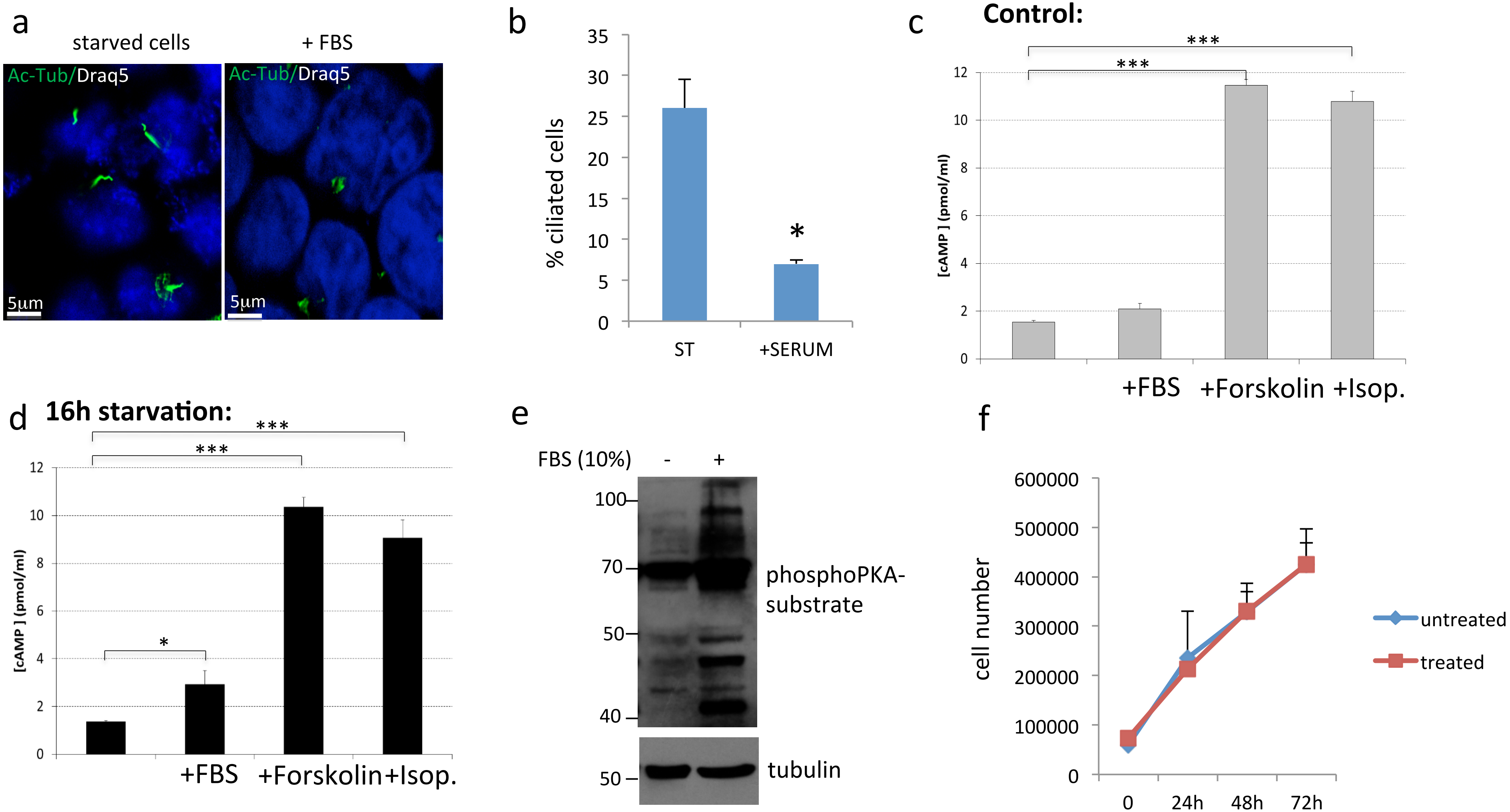

Supplementary Figure 1. Effect of fetal bovine serum and indicated cAMP-mobilizing agents on cellular cAMP levels and cilia resorption. (**a**) HEK293 cells were serum-deprived for 36h and then left untreated (CNT) or stimulated for 6 hours with fetal bovine serum (FBS) and doubly stained for acethylated tubulin and Draq5. (**b**) Cumulative data from 3 independent experiments are shown. *p<0.05 (students t test) versus control (ST).(**c-d**) HEK293 cells grown in the presence (**c**) or absence (**d**) of fetal bovine serum (FBS; starvation for 16h) were treated with fetal bovine serum (10%), 20 mM Forskolin or 100 nM Isoproterenol for 20 min. After treatments cells were lysed and subjected to cAMP measurements using the *Direct cAMP Enzyme Immunoassay Kit* following the instructions of the manufacturer. Cumulative data from 3 independent experiments are shown (±SEM). Paired student's t-test was used to evaluate statistical significance. Confidence level: *P<0.05, **P<0.01, ***P<0.001.(**e**) Starved cells were left untreated or stimulated with FBS for 3 hours. Lysates were immunoblotted with anti-phosphoPKA substrates antibodies. A representative set of experiments is shown. (**f**) Starved cells, left untreated or treated with FSK for 3hours, were washed twice with PBS1X and then grown in FBS (10%)- supplemented medium. Cells were harvested at the indicated time points and counted. Cumulative data from 4 independent experiments made in triplicate are shown.

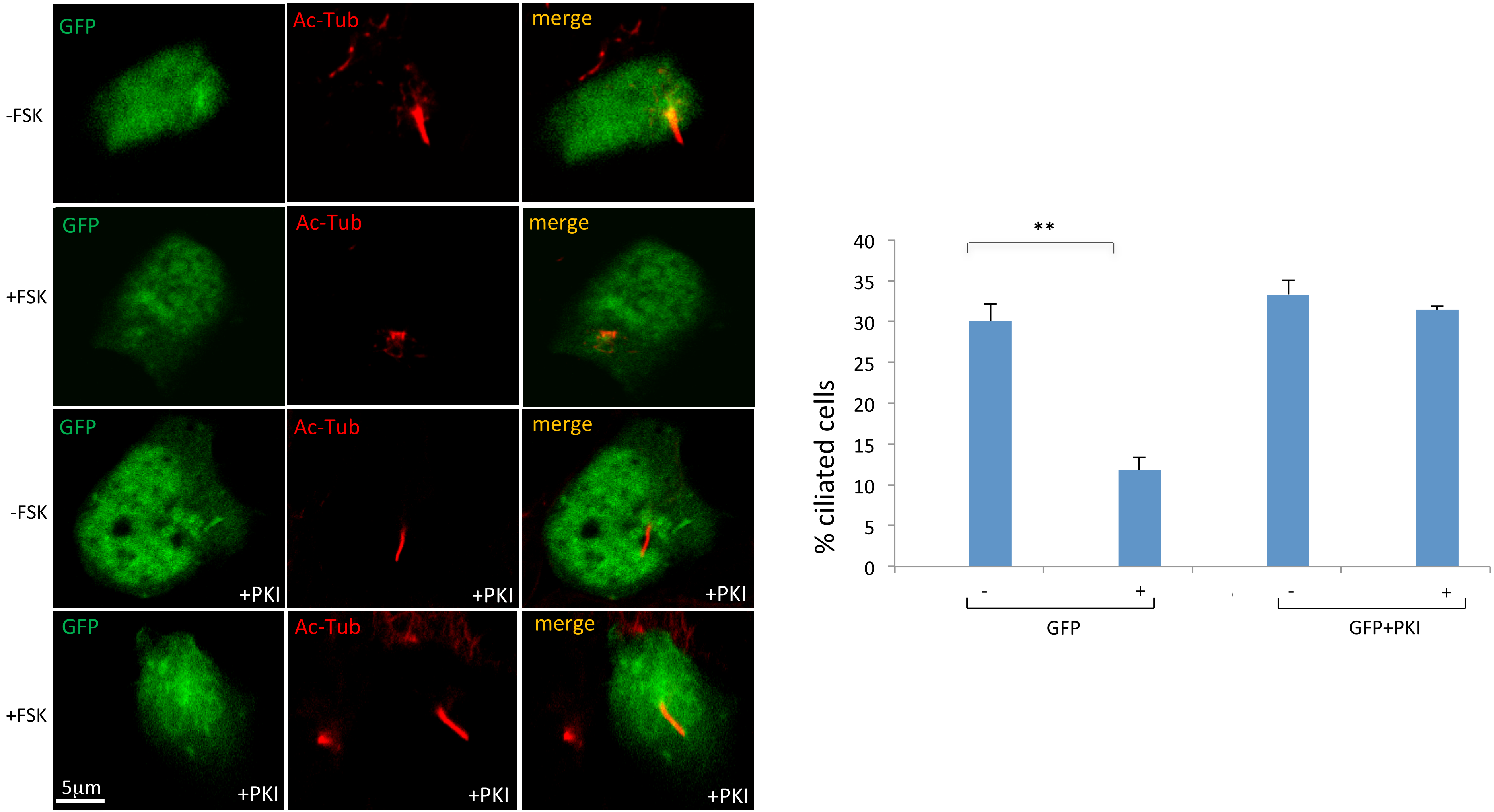

Supplementary Figure 2. PKI expression prevents primary cilium resorption. HEK293 cells were serum deprived for 24 hour, transiently (eight hours) cotransfected with PKI and GFP vectors, and then treated with FSK (40µM 3h). Fixed cells were immunostained for acetylated tubulin. Cumulative data from 3 experiments are shown. For each experimental group a minimum of 55 GFP positive cells was averaged. **P<0.01.

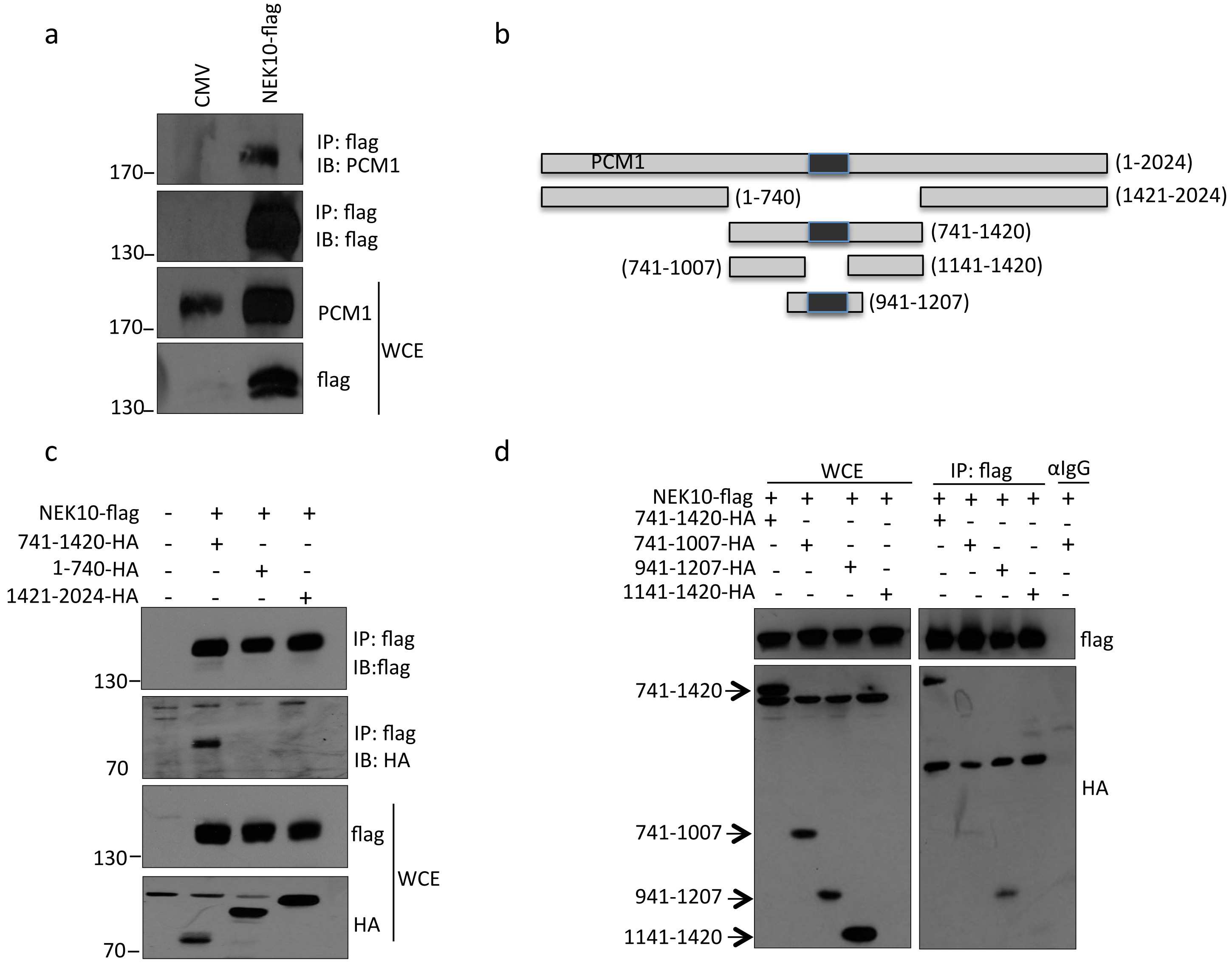

Supplementary Figure 3. Identification of NEK10 binding domain on PCM1. (**a**) Lysates from cells transiently expressing NEK10-flag were immunoprecipitated with anti-flag antibodies. The precipitates and whole cell lysates (WCE) were immunoblotted with anti-flag and anti-PCM1 antibodies. (**b**) Schematic diagram of the HA-tagged PCM1 deletion mutants. (**c-d**) Lysates from Cells transiently expressing NEK10-flag and PCM1 deletion mutants were immunoprecipitated with anti-flag antibodies. The precipitates and lysates were immunoblotted with the indicated antibodies.

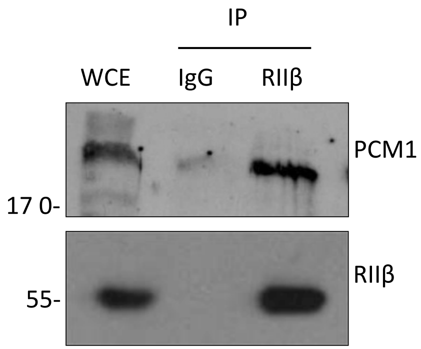

Supplementary Figure 4. PCM1 forms a complex with RIIβ. RIIβ was immunopurified from whole HEK293 cell extracts (WCE). IgG were used as control. The precipitates and lysates were immunoblotted with anti-PCM1 and anti-RIIβ antibodies.

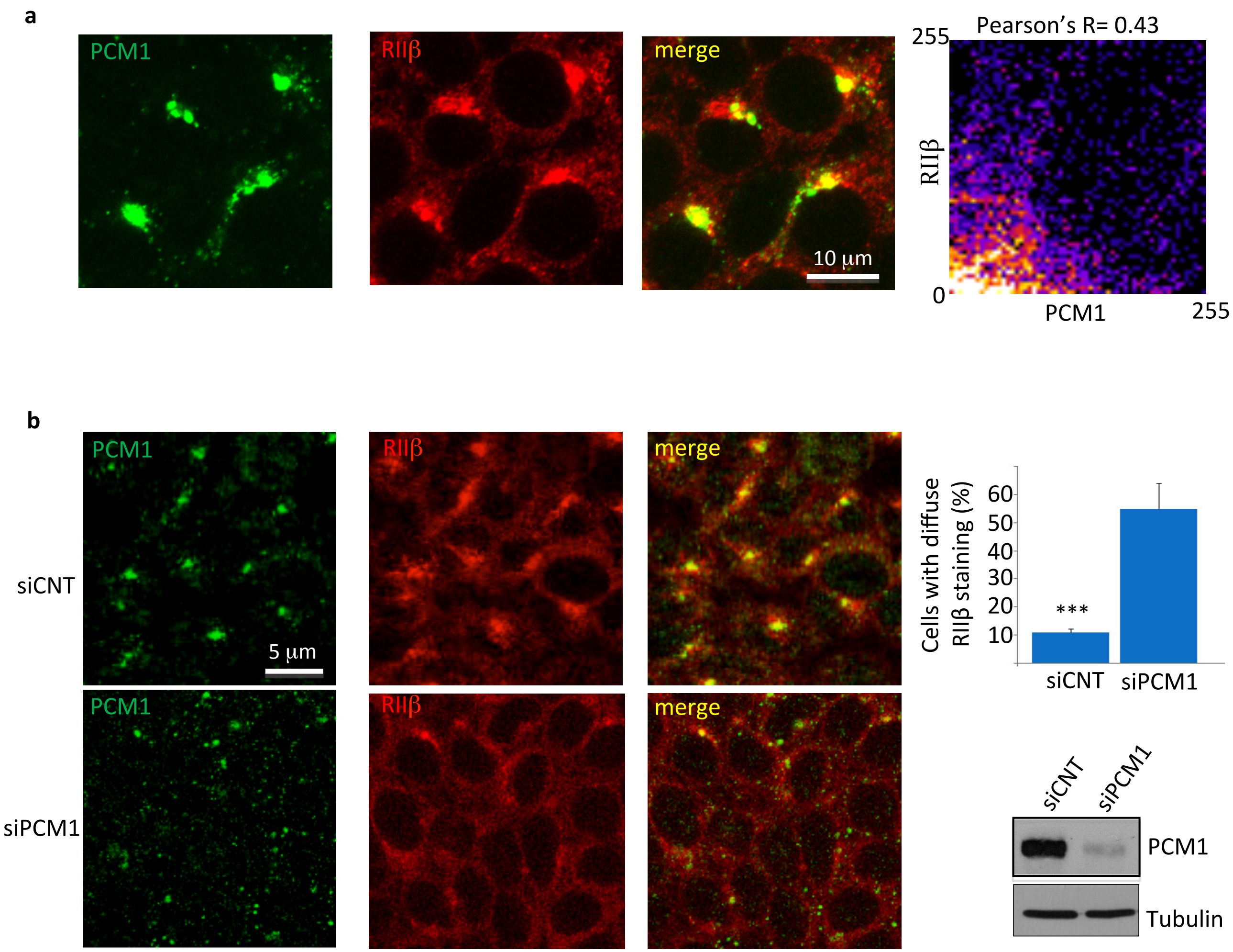

Supplementary Figure 5. PCM1 is required for RIIβ localization at pericentriolar matrix. (**a**) Cells were fixed and doubly immunostained for RIIβ and PCM1. A merge composite and Pearson's coefficient are shown. (**b**) Cells were transfected with siRNA targeting PCM1 or with control siRNAs, fixed and doubly immunostained for RIIβ and PCM1. Upper right panel. Cumulative data of the effects of siPCM1 on delocalization of RIIβ signal from pericentriolar matrix are shown. Lower right panel. The levels of PCM1 in transfected cells were analyzed by immunoblot analysis. ***$p<0.001$.

a

b

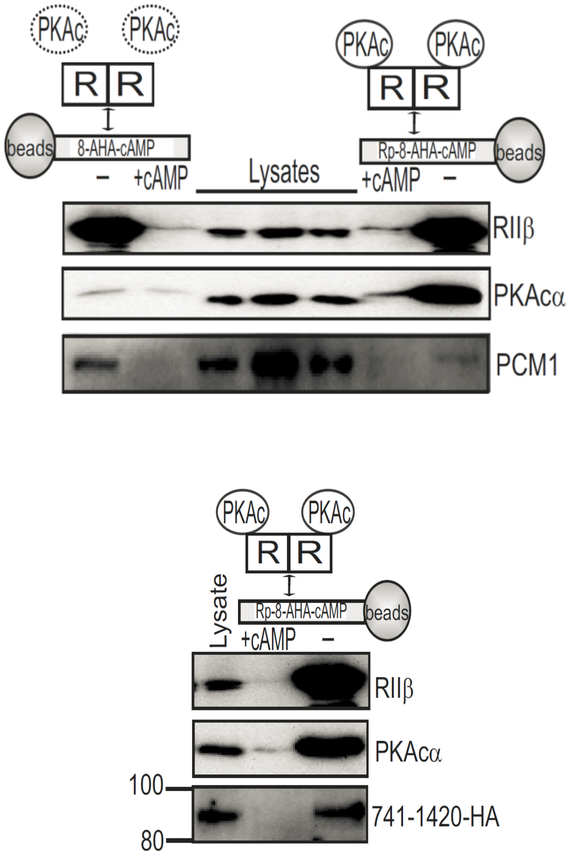

Supplementary Figure 6. cAMP precipitation of PCM1/PKA complexes. (**a**) Cell lysates were subjected to cAMP purification assay, using either 8-AHA-cAMP- or Rp-8-AHA-cAMP-coupled beads. Purified complexes were immunoblotted for PCM1, PKAcα and RIIβ. In the control, a molar excess of cAMP (5 mM) was added to the lysate. (**b**) Lysates from cells transiently expressing the HA-tagged PCM1 contruct were subjected to cAMP purification assay. Purified complexes were immunoblotted for HA, PKAcα and RIIβ. In the control, a molar excess of cAMP (5 mM) was added to the lysate.

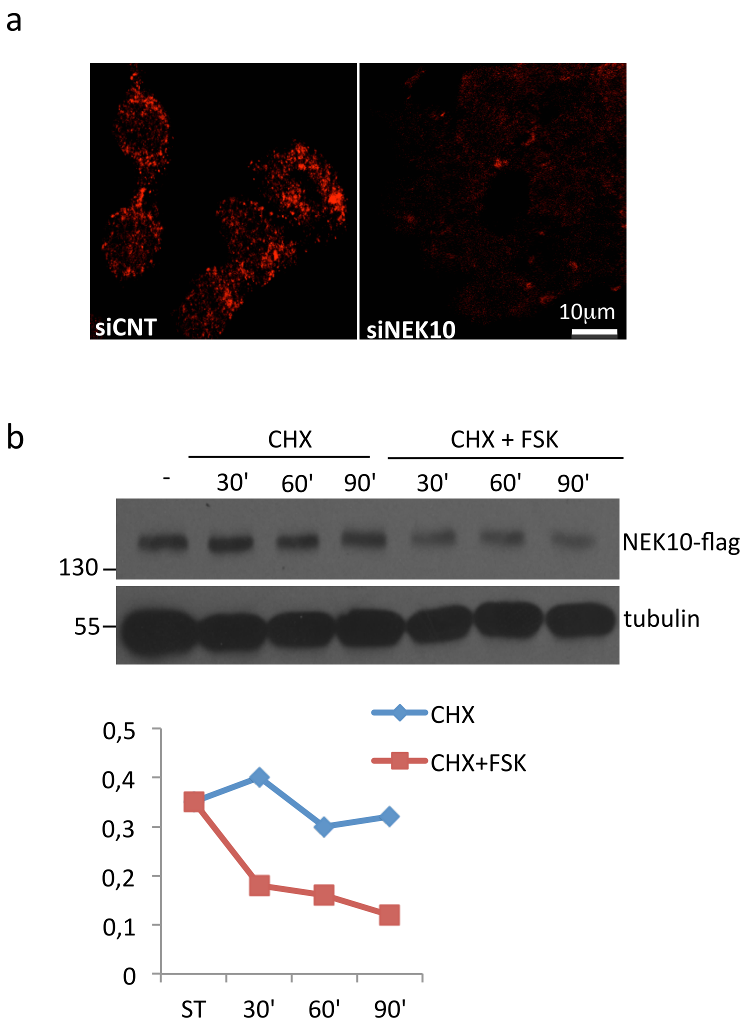

Supplementary Figure 7. cAMP controls NEK10 stability **(a)** The levels of NEK10 in siRNA-transfected cells were monitored by immunostaining. **(b)** Cells transiently transfected with NEK10-flag were incubated with cycloheximide and left untreated or stimulated with FSK (40μM). Cells were harvested at the indicated time points and lysed. Lysates were immunoblotted with anti-flag and anti-tubulin antibodies. A quantitative analysis is shown in the lower histogram.

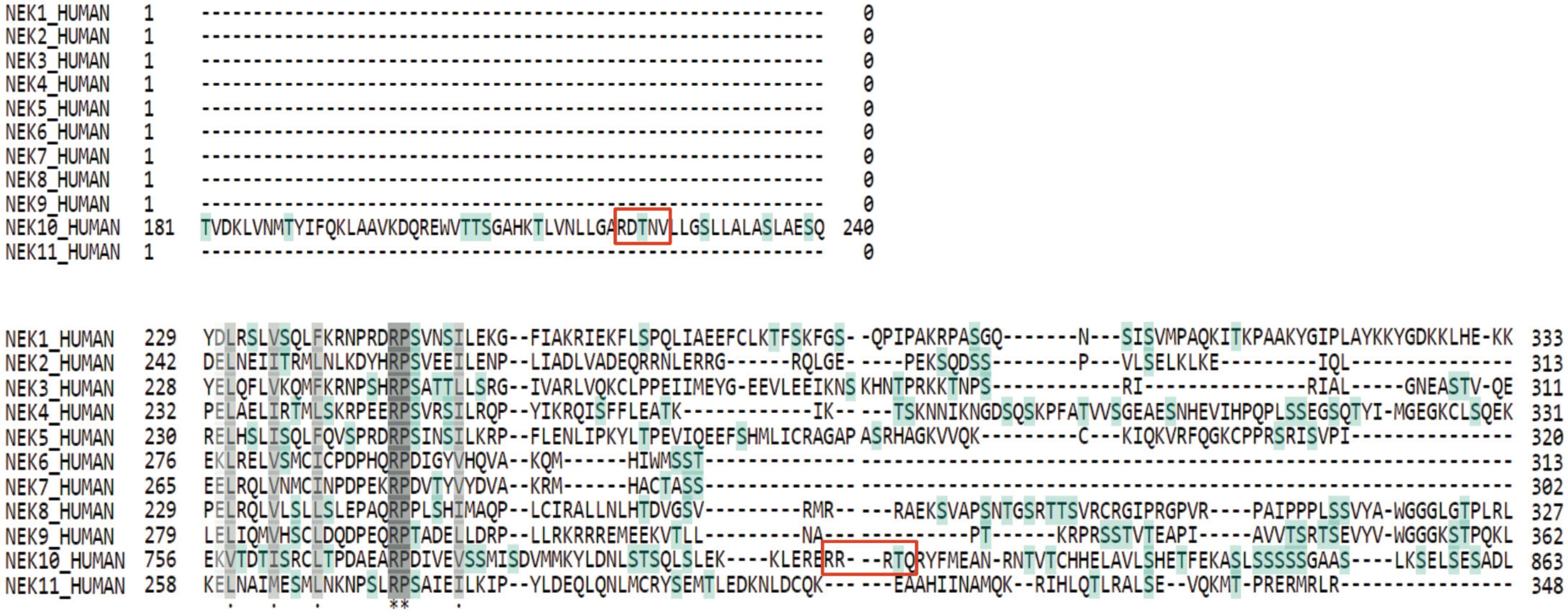

Supplementary Figure 8. Protein sequence alignment of NEK family members. The predicted PKA phosphorylation sites (T223 and T812) of NEK10 are indicated in red.

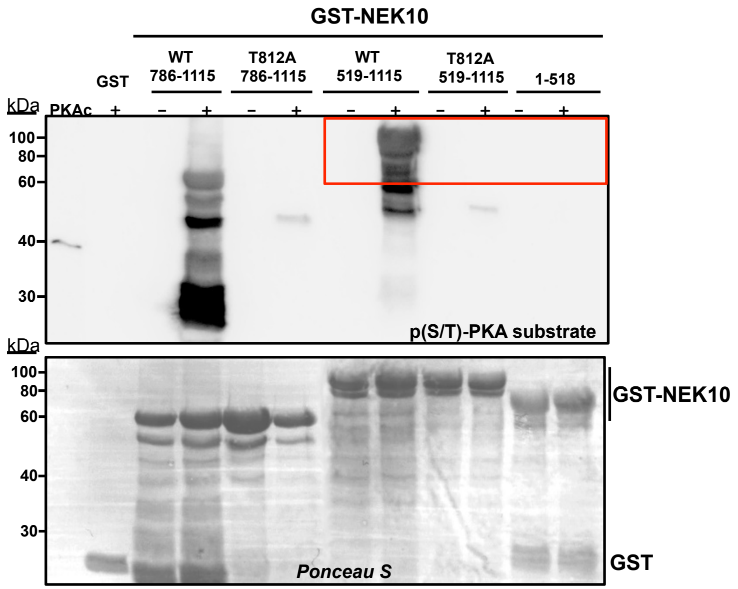

Supplementary Figure 9. Recombinant and GST-fused NEK10 proteins were used as substrates for *in vitro* phosphorylation assays using recombinant and hexahistidin-tagged PKAc. GST-immobilized fusion proteins were analyzed in immunoblotting experiments with an anti-phospho-(K/R)(K/R)X(S*/T*) specific antibody. Shown is a representative result from n=3 independent experiments. Red frame indicates the parts used for the main figure.

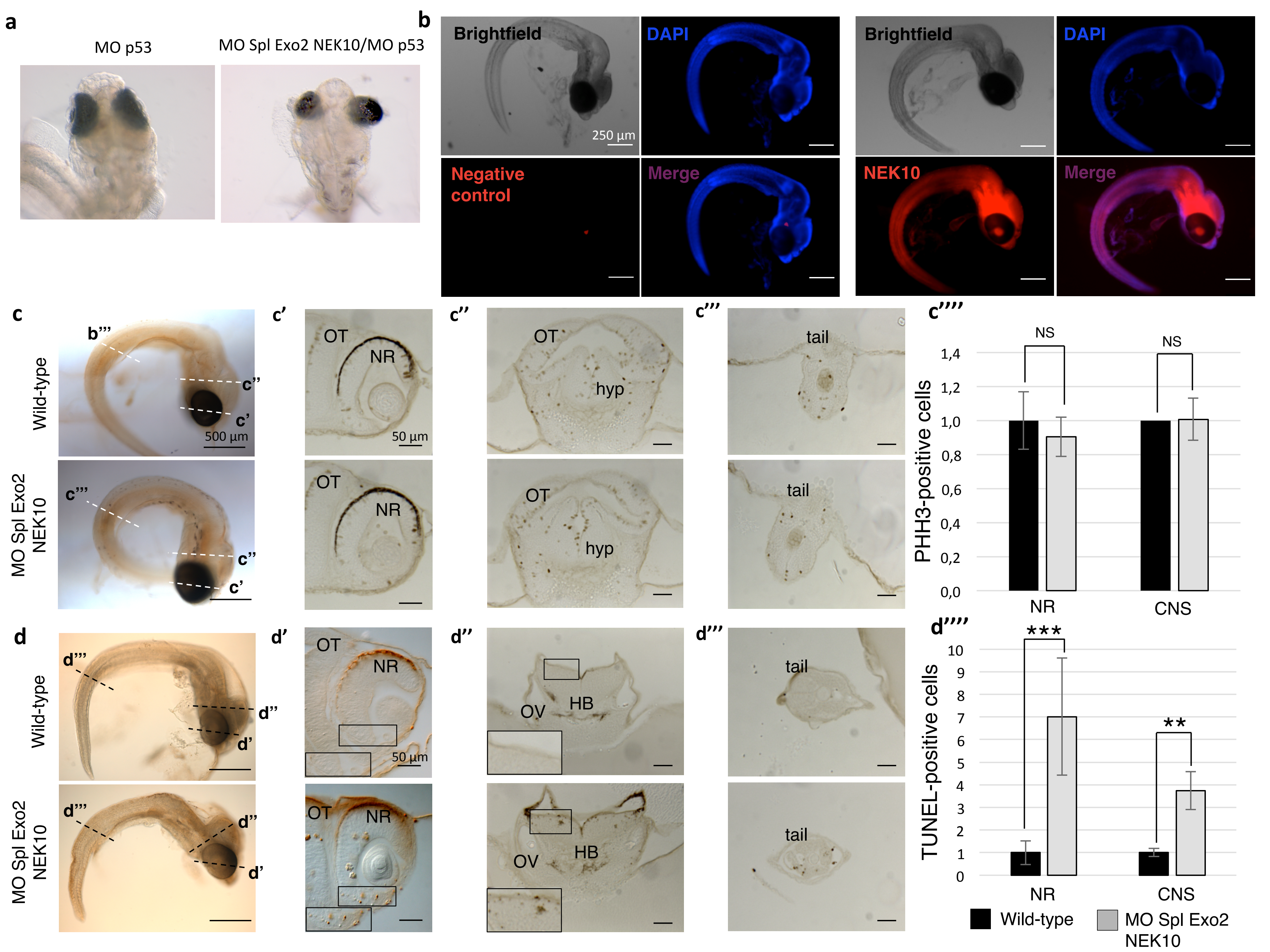
a
MO p53
MO Spl Exo2 NEK10/MO p53
b
Brightfield
DAPI
250 µm
Negative control
Merge
Brightfield
DAPI
NEK10
Merge
c
Wild-type
b'''
c''
c'
500 µm
MO Spl Exo2 NEK10
c'''
c''
c'
c'
OT
NR
50 µm
c''
OT
hyp
c'''
tail
c''''
NS
NS
PHH3-positive cells
1,4
1,2
1,0
0,8
0,6
0,4
0,2
0,0
NR
CNS
d
Wild-type
d'''
d''
d'
MO Spl Exo2 NEK10
d'
OT
NR
50 µm
d''
OV
HB
d'''
tail
d''''
***
**
TUNEL-positive cells
10
9
8
7
6
5
4
3
2
1
0
NR
CNS
Wild-type
MO Spl Exo2 NEK10

Supplementary figure 10. Expression analysis of NEK10 in Medaka embryo. (**a**) Stereomicroscopic images of MO p53 and MO Spl Exo2 NEK10/ MO p53 injected Medaka larvae, at stage 40. No modifications of the phenotype was observed, supporting the high specificity of MO Spl Exo2 NEK10 phenotype. (**b**) Representative Nek10 immunostaining in the whole embryo. Nek10 resulted ubiquitously expressed with high level in the central nervous system (CNS). (**c**) Bright-field microscopy images of lateral views of control-injected and morphant embryos stained for phosphorylated histone H3 (PHH3), a specific marker for cells in the M-phase. (**c'-c'''**) PHH3 vibratome sections of phenylthiourea-treated control-injected and morphant embryos at stage 32. No alteration in the number of proliferating cells (brown spots) in morphant embryos was detected in comparison control-injected embryos. NR=neural retina; OT=optic tectum; TE=telencephalon; hyp=hypothalamus. (**c''''**) Quantification of PHH3-positive cells in the neural retina (NR) and in the central nervous system (CNS) of control-injected (black bars) and MO Spl Exo2 NEK10-injected (grey bars). (**d**) Frontal vibratome sections of TUNEL stained control-injected, morphant embryos. A significant increase of cell death (brown spots) in the whole embryos was observed compared with control-injected embryos. (**d'-d'''**) TUNEL vibratome sections of phenylthiourea-treated control-injected and morphant embryos at stage 32. Apoptotic cells are visible as brown spots. NR=neural retina; OV=optic vescicle; HB=hindbrain. (**d''''**) Quantification of TUNEL-positive cells in the neural retina (NR) and in the central nervous system (CNS) of control-injected (black bars) and MO Spl Exo2 NEK10-injected (grey bars). Student's t-test ** $p \leq 0.01$, ***$p \leq 0.005$.

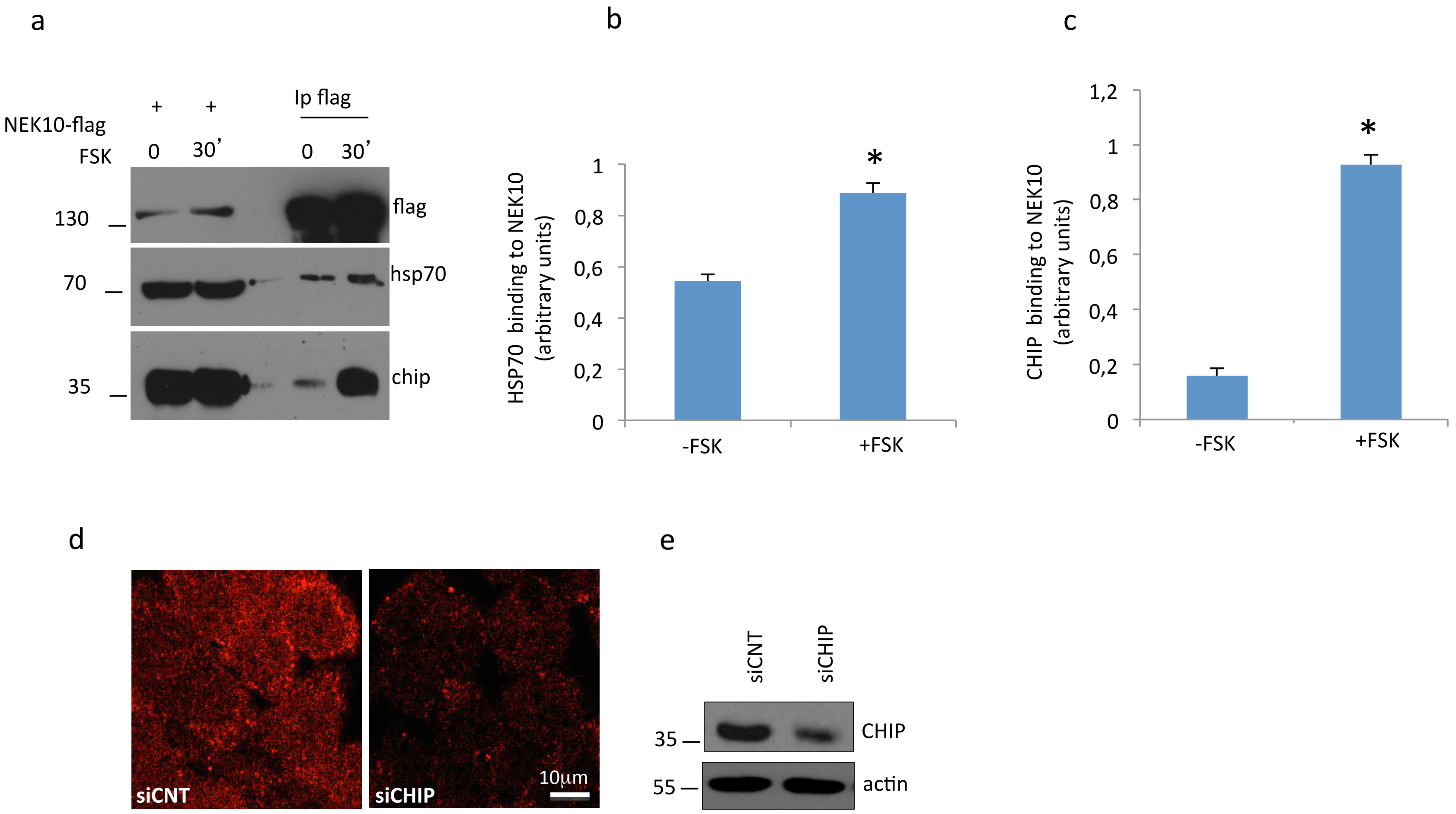

Supplementary Figure 11. (a) Cells were were transfected with NEK10-flag vector, serum deprived for 24 hours and left untreated or stimulated with FSK. Lysates were immunoprecipitated with anti-flag. The precipitates and lysates were immunoblotted for HSP70 and CHIP. (**b-c**) Cumulative data of NEK10 binding to HSP70 (**b**) or to CHIP (**c**). A mean value ± SEM from three independent experiments is shown. *p<0.05 versus control (-FSK). (**d-e**) The levels of CHIP in siRNA-transfected cells were monitored by immunostaining (**d**) and immunoblot (**e**) analyses.

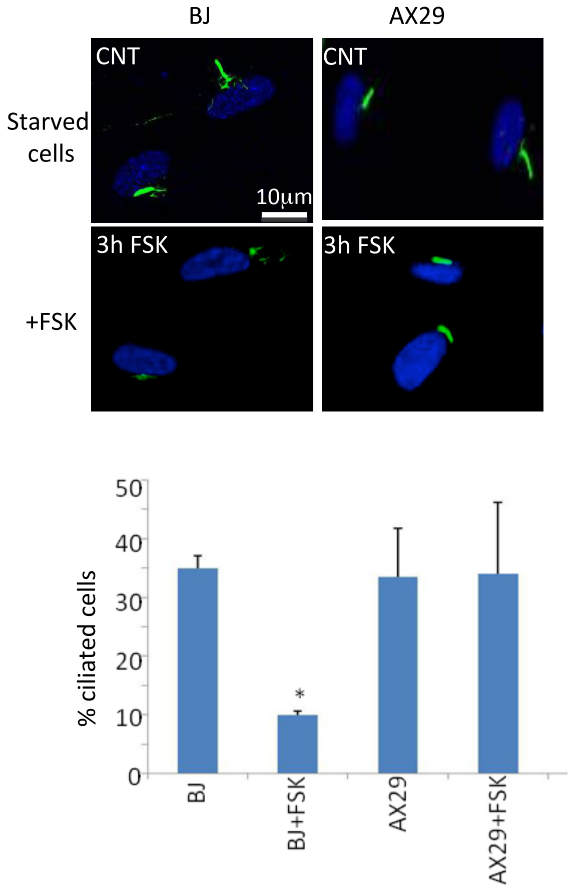

Supplementary Figure 12. Effects of cAMP stimulation on primary cilium in healthy and diseased human fibroblasts. Skin fibroblasts from healthy volunteers (BJ) and from SCAR16 (AX29) patients were serum deprived for 48h and treated with FSK (80 µM/6h). Cells were fixed and stained for acetylated tubulin. Nuclei were stained with Draq5. (**b**) Cumulative data from 4 independent experiments are shown. *$p<0.05$ versus control (BJ, -FSK) and AX29 (±FSK).

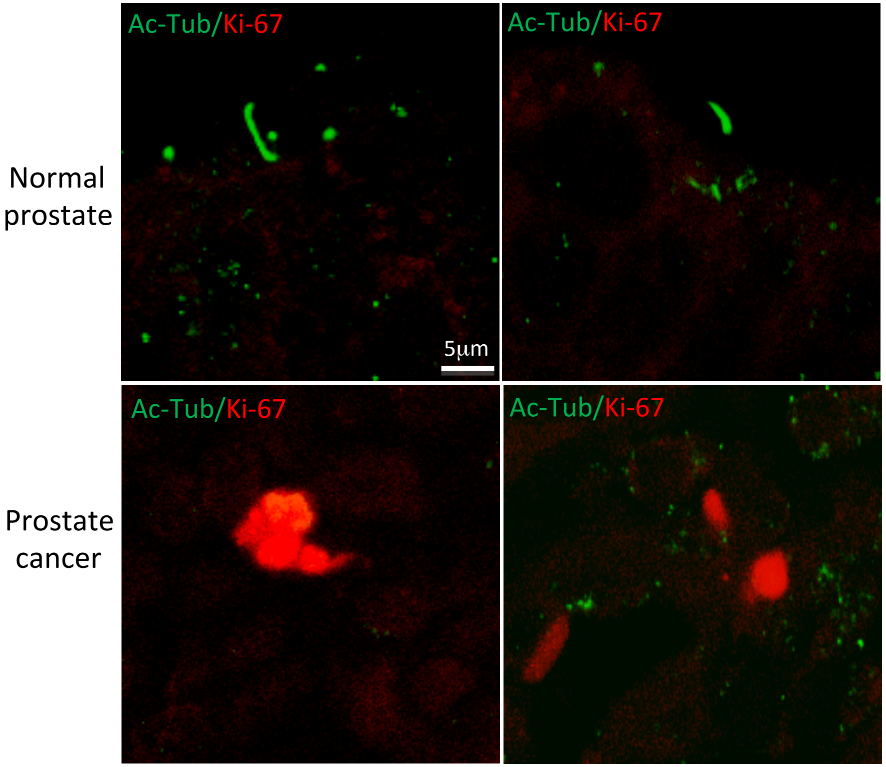

Supplementary Figure 13. Loss of primary cilium in high-grade prostate cancer. Immunohistochemistry for acetylated tubulin and Ki-67, a proliferative marker, on tissue sections from normal prostate and from cancer tissues. A representative set of images is shown.

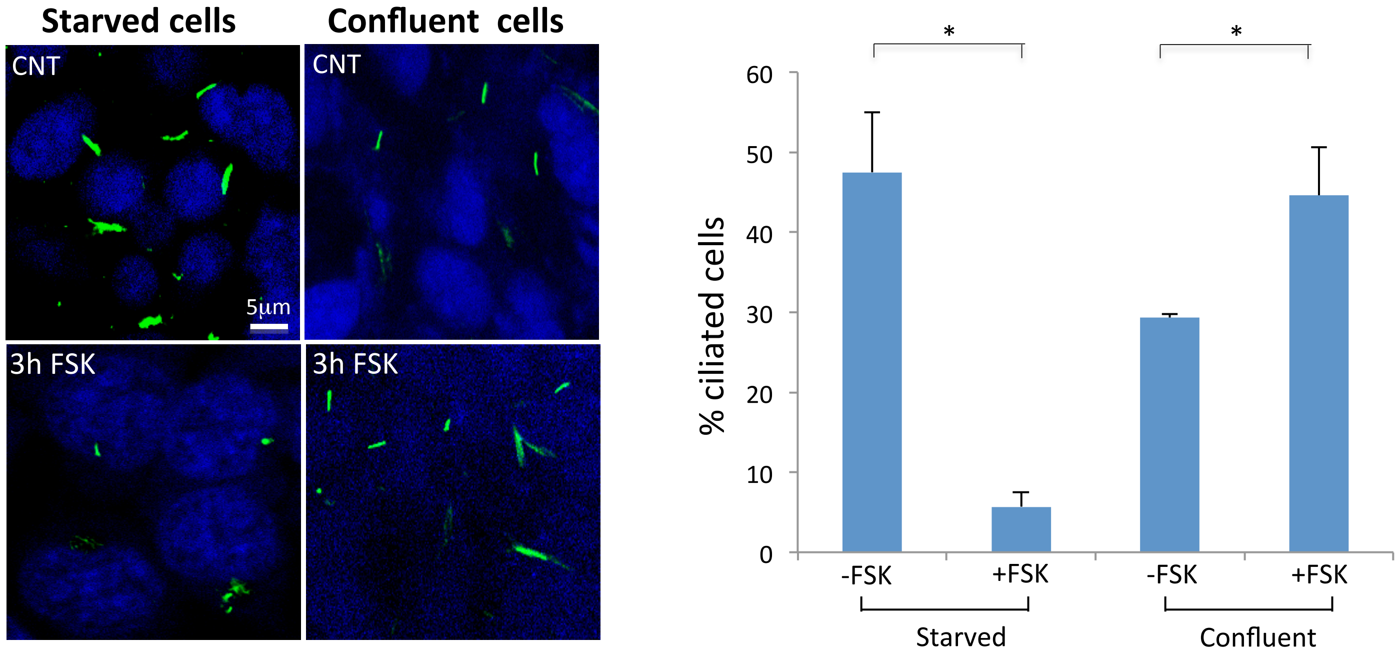

Supplementary Figure 14. Effects of cAMP on primary cilium in confluent and in serum-deprived cells. HEK293 cells grown to confluency or serum deprived for 36 hours were treated with FSK (40μM/3h) and immunostained for acetylated tubulin. Nuclei were stained with Draq5. Cumulative data from 3 independent experiments are shown. For each group a minimum of 65 cells/experiment was averaged. *$p<0.03$ (starved cells); *$p<0.04$ (confluent cells).

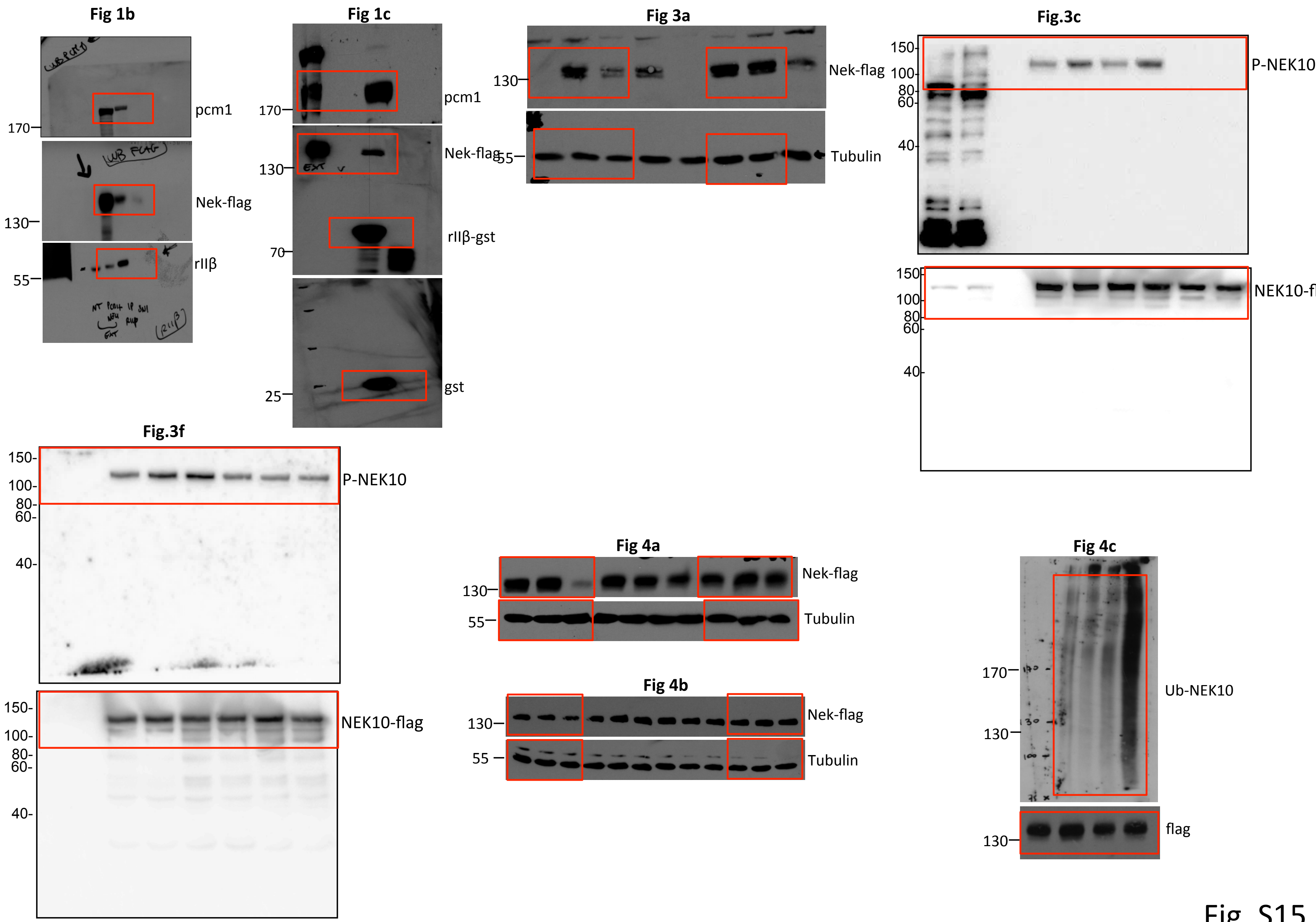

Fig 1b
pcm1
Nek-flag
rIIβ
170
130
55
Fig 1c
pcm1
Nek-flag
rIIβ-gst
gst
170
130
70
25
Fig 3a
Nek-flag
Tubulin
130
55
Fig.3c
P-NEK10
NEK10-f
150
100
80
60
40
Fig.3f
P-NEK10
NEK10-flag
150-
100-
80-
60-
40-
Fig 4a
Nek-flag
Tubulin
130
55
Fig 4b
Nek-flag
Tubulin
130
55
Fig 4c
Ub-NEK10
flag
170
130

Fig. S15

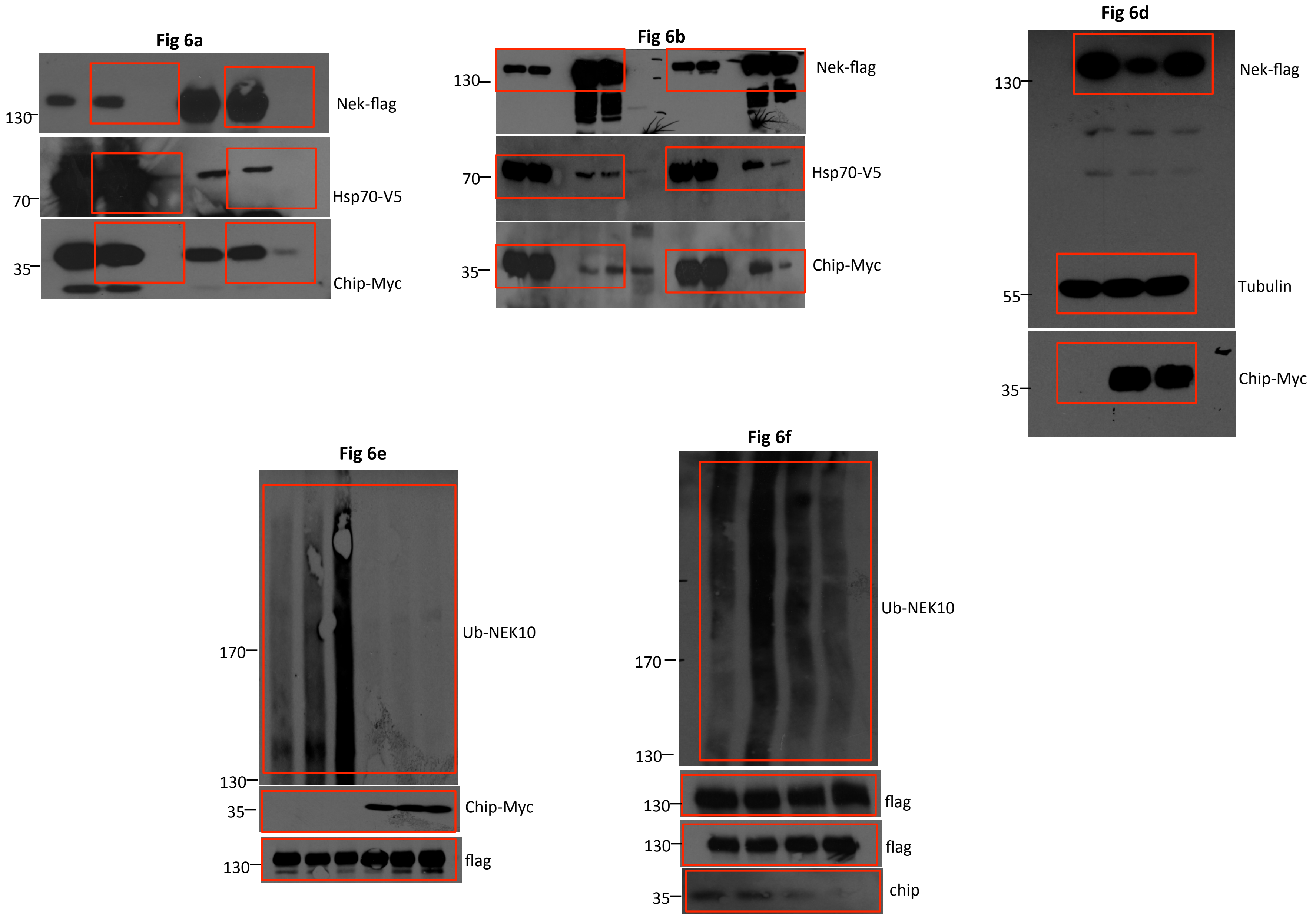

Supplementary Figure 15. Full scans of western blots presented. The area used as data in the main figures are indicated by the red boxes. Molecular weight markers are included.
