# Supplementary material for: Counterregulation of cAMP-directed kinase activities controls ciliogenesis
Source: Nat Commun. 2018 Mar 26;9:1224. doi: 10.1038/s41467-018-03643-9 (PMC5964327; doi:10.1038/s41467-018-03643-9)
Supplement: Supplementary file 2 — Description of Additional Supplementary Files(PDF 164 kb) [file 41467_2018_3643_MOESM2_ESM.pdf]

Description of Additional Supplementary Files

File Name: Supplementary Movie 1
Description: Localization of NEK10 at primary cilium.
